# Nucleic acid-loaded poly(beta-aminoester) nanoparticles for cancer nano-immuno therapeutics: the good, the bad, and the future

**DOI:** 10.1007/s13346-024-01585-y

**Published:** 2024-05-03

**Authors:** J. Rodrigo Magaña Rodriguez, Marta Guerra-Rebollo, Salvador Borrós, Cristina Fornaguera

**Affiliations:** Grup d’Enginyeria de Materials (Gemat), Institut Químic de Sarrià (IQS), Universitat Ramon Llull (URL), Barcelona, 08017 Spain

**Keywords:** Poly(beta aminoesters), Cancer immunotherapies, Polymeric nanoparticles, Nucleic acids therapeutics, Controlled delivery

## Abstract

Immunotherapy has emerged as a promising approach to cancer treatment, offering improved survival rates and enhanced patients’ quality of life. However, realizing the full potential of immunotherapy in clinical practice remains a challenge, as there is still plenty of room for modulating the complexity of the human immune system in favor of an antitumor immunogenicity. Nanotechnology, with its unique properties, holds promise in augmenting the efficacy of cancer immunotherapies in biotherapeutic protection and site- and time-controlled delivery of the immune modulator biologicals. Polymeric nanoparticles are promising biomaterials among different nanocarriers thanks to their robustness, versatility, and cost-efficient design and production. This perspective paper overviews critical concepts in nanometric advanced delivery systems applied to cancer immunotherapy. We focus on a detailed exploration of the current state of the art and trends in using poly(beta-aminoester) (pBAE) polymers for nucleic acid-based antitumor immunotherapies. Through different examples of the use of pBAE polymers reported in the literature, we revise the main advantages these polymers offer and some challenges to overcome. Finally, the paper provides insights and predictions on the path toward the clinical implementation of cancer nano-immunotherapies, highlighting the potential of pBAE polymers for advancements in this field.

**Graphical abstract:**

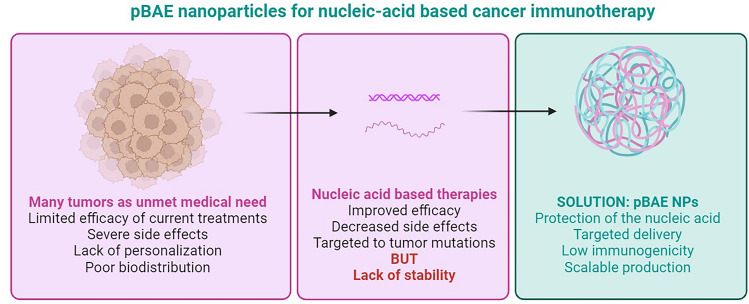

## Introduction

 Cancer continues to stand out as a prominent contributor to global mortality rates, particularly in developed nations—some tumor types, such as lung and pancreatic, exhibit devastating mortality rates and poor survival prospects. Prognoses, however, have experienced a gradual improvement with the advent of cancer immunotherapies in clinical practice. These innovative treatments can counteract the immune suppression within the tumor microenvironment, allowing the ubiquitous immune system to deplete cancerous cells [1, 2]. Within the spectrum of potential immunotherapies, immune checkpoint inhibitors (ICI), such as anti-PD-1 antibodies, have revolutionized the management of many cancer types, becoming the standard of care for various solid tumors, such as melanoma and some lung cancer subtypes [3, 4]. Even though up to ten different ICI-based therapies have been FDA accepted since the first approval of Ipilimumab in 2011, high ratios of patients (i.e.,>75% of lung cancer) do not respond to these treatments due to primary or acquired resistances [5, 6]. Consequently, realizing the complete potential of immunotherapy in clinical practice remains an ongoing pursuit.

The reasons behind the low penetration of immunotherapies into clinical practices are diverse. Among the main reasons is the short half-life of biological drugs (i.e., proteins or nucleic acids), often requiring protective mechanisms from nucleases and proteases. In addition, premature clearance is commonly experienced due to the immune system’s erroneous recognition of the biologicals. Finally, poor biodistribution prevents the full potential of the biologicals due to their accumulation in non-target organs, which, in addition to reducing efficacy, eventually produces side effects. In this context, nanomedicine has been widely described as the game-changer technology to augment the safety and effectiveness of cancer immunotherapies [6].

A couple of decades ago, researchers introduced the application of sub-micron size particles to control the delivery of active principles, the so-called nanomedicine field. The market witnessed the debut of the initial nanomedicines, with a primary focus on enhancing cancer chemotherapy. One notable example is Doxil, a liposome that encapsulates doxorubicin [7]. Since then, examples of nanomedicines for drug delivery are vast, with lipid and polymeric nanocarriers as the primary technologies.

Polymeric nanoparticles, constituted by biodegradable polymers, are considered an essential platform for controlled drug and gene delivery to specific organs and cells in the body. By controlling their design, they are advantageous among lipid and other competitors in terms of capacity to encapsulate more than one active, decreased immunogenicity, and modulable properties. The clinical use of polymeric nanoparticles lays its foundations in the use of previously approved polymers for human use in other types of medical devices such as sutures and drug depots [8], as poly (lactic-co-glycolic) acid (PLGA) [9, 10] and poly (ethylene glycol) (PEG) [11].

**Fig. 1:**
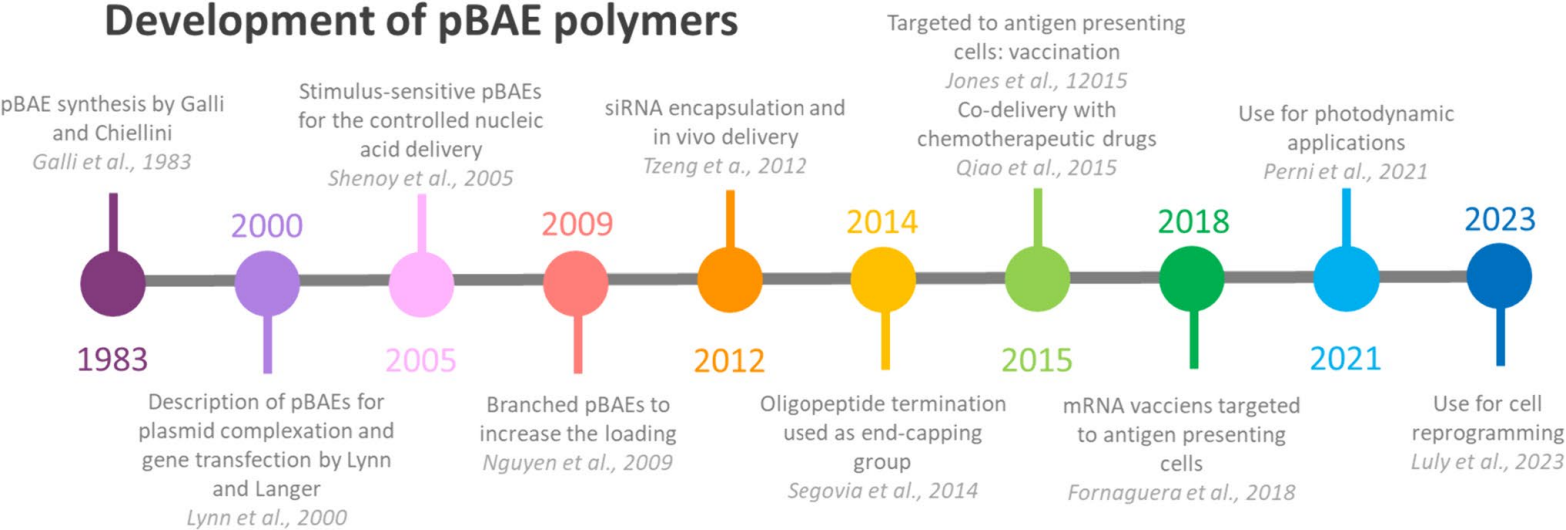
History of pBAEs development. Schematic representation of the main milestones in the history of the research referred to the development of pBAEs that enabled their current use for immune therapeutic applications

Among the different promising polymers to enter clinical trials, poly(beta aminoesters) – pBAEs, recognized for nucleic acids encapsulation, are expected to arrive at clinics shortly, not only for cancer immunotherapy but also for many other unmet medical needs, such as cartilage regeneration or rare diseases gene edition treatment [12–16]. pBAEs were first described in 2000 by Lynn and coworkers [17], as the second generation of nucleic acid cationic carriers, after the first-generation carriers demonstrated some toxicity [18, 19]. Only ten years later, their potential application for immune and tumor gene modulation as advanced therapeutics was rapidly and widely recognized [20–25]. Twenty years after their initial description, their immunotherapeutic efficacy has been demonstrated preclinically (Fig. 1) [26].

## pBAE polymers for polynucleotide entrapment: chemical versatility and robustness in design

Polynucleotide encapsulation generally takes place due to electrostatic interaction between the negatively charged pyridinic bases and positively charged matter. Examples of oligonucleotide encapsulation materials include (bio) polymers, cationic lipids, and nanoparticles. Even though a strong electrostatic complexation is present, these carriers loaded with genetic material require a myriad of chemical and physical properties that eventually will lead to improved bioavailability, transfection efficiently, and reduced toxicity and bioaccumulation. Through this perspective we do not aim in covering all of these physicochemical properties but rather give a broad overview. The interested reader might refer to other more technical seminal reviews [27, 28].

Indeed, the design of the cationic carrier is a pivotal factor in gene therapies, influencing both transfection efficiency and toxicity. Regrettably, optimizing these carriers often presents a dilemma: as transfection efficiency improves, toxicity tends to rise, and conversely, less toxic compounds usually exhibit lower transfection efficiency. An illustrative example is transfection agent polyethylene imine (PEI, jet-PEI), which yields among the highest transfection rates among all non-viral gene carriers [29]. Attempts to mitigate this toxicity by the addition of polyethylene glycol (PEG), a hydrophilic and biocompatible polymer, into PEI structure come with trade-offs: While PEGylation improves cell viability, it comes at the expense of reduced cellular uptake, compromised endosomal escape, and consequently, lower transfection efficiency. Less cytotoxic polyelectrolytes, such as poly-lysine also can reduce cytotoxicity, but again at the expense of lower transfection efficiency.

Notably, high molecular weight polymers generally exhibit superior DNA/RNA binding, cellular uptake, and transfection efficiency. Conversely, low molecular weight polymers are less cytotoxic and proficient in efficiently unpacking polynucleotides post-transfection [30–32]. Striking the right balance between these factors becomes a critical aspect of optimizing cationic carriers for effective and safe gene delivery. Striving to achieve an optimal balance, Reineke et al. innovatively crafted short, linear, neutral-cationic copolymers by combining biocompatible carbohydrates with short PEI, resulting in the formation of poly(glycoamidoamine)s (PGAAs). Notably, the polyplex transfection rates are notably elevated owing to the PEI chain, while the protective carbohydrate block and the lower molecular weight concurrently mitigate cytotoxicity. PGAA usually prepared in excess polymer yields nanoparticles with positive surface charge, enhancing interactions with negatively charged proteins on the cell wall and facilitating endocytosis. Through systematic variations in carbohydrate type, the number of charges, and their sequence in PGAA, the study revealed that transfection efficiency is significantly influenced by various factors [33–35]. These include the augmentation of charge in response to endosomal pH, endosomal escape, and the binding strength of the polymer to the oligonucleotide [35].

These examples just give us a glimpse of the intricacies and endless possibilities when designing a new gene-based therapy. In this sense carrier platforms with high chemical modularity (i.e. can be easily modified or varied) represent tremendous advantages towards modulating the desired biochemical response and designing new delivery systems.

Poly (beta aminoester) – pBAE polymers are generally synthesized by simple Michael addition between diacrylates and primary amines. Because of this relatively simple synthetic methodology and the broad spectrum of available monomers (commercially available or synthetically made), pBAEs have a high chemical versatility as side chains are selected to meet quality criteria. More than 2350 linear pBAE variants have different backbone and ramification (i.e. attachment of lateral side chains) groups [14, 26, 36].

**Fig. 2:**
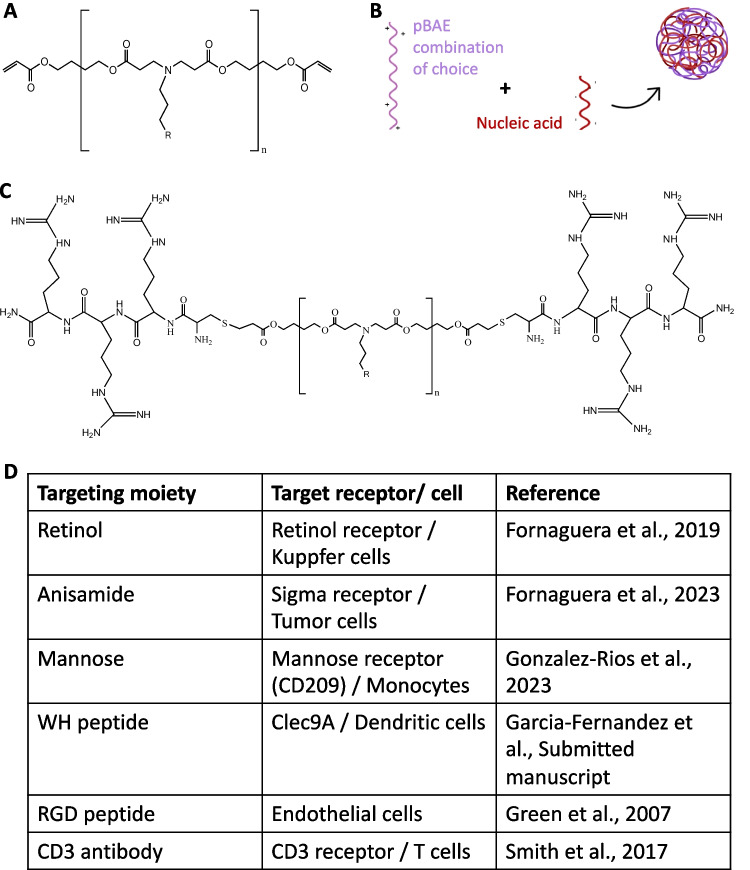
pBAE nanoparticles composition summary. **A** Chemical structure of the pBAE polymer general backbone, including the end-oligopeptide modification. *n* = 6–8 repetitions. R = lateral chain functionalization, where the targeting moieties and also functional groups can be added. **B** Formation of pBAE nanoparticles thanks to the cationic polymer complexation with anionic nucleic acids. **C** Chemical structure of OM-pBAE. **D** Table summarizing some of the main targeting strategies used for pBAE active targeting

pBAEs’ structure (Fig. 2A) offers several advantages concerning other carriers thanks to their pH-dependent charge, which promotes binding to negatively charged nucleic acids at slightly acidic pH (Fig. 2B), its biocompatibility, biodegradation and high transfection efficacy [37–39]. This strong electrostatic interaction leads to the formation of nanoparticles with minimal energy input (i.e., manual pipetting or automatized microfluidics mixing). Consequently, the encapsulation efficiency, or rigorously, the entrapment efficiency, and the loading capacity of pBAE nanoparticles are commonly very high, with values above 70%. Indeed, mainly electrostatic interactions leads to the entrapment of polynucleotide chains via the formation of polyelectrolyte complexes, however, other molecular functionalities are introduced in the pBAE backbone, such as hydrophobic moieties to increase colloidal stability and biocompatibility. The structure and properties of these, so called polyplexes, generally depends on external cues, such as pH, ionic strength and on internal ones such as polymer architecture, and molecular weight and type polynucleotide [27]. For example, we have systematically observed that mRNA nanoparticles are smaller than those prepared with DNA. Regarding its internal structure, we have previously observed the relative homogenous distribution of polynucleotides in spherical-like pBAE nanoparticles using fluorescence resonance energy transfer (FRET) [40].

Chemically, the contribution of the spacer length, side chain lengths, lipophilic/hydrophilic balance, and polymer chain end is usually correlated with transfection efficiency and biocompatibility. In this sense, the molecular design of the pBAE backbone can be adapted to different applications (see Table 1 for details of other pBAE structures). For starters, higher hydrophobicity in the backbone has been shown to improve the transfection efficiency of pBAE nanoparticles in some cases [41]. Amine-terminated polymers usually cause higher transfection compared to those terminated in acrylate [42, 43]. This is probably due to the higher positive charge and its interaction with cellular membranes. Regarding biocompatibility issues, linear pBAEs are preferred, allowing better control of resulting nanoparticle size when scaling up the formulation and encapsulating more polynucleotides [44–46]. On the other hand, highly branched pBAEs are more stable and robust, allowing a higher transfection efficiency due to the higher density of cationic charges that also facilitates the encapsulation of more molecules of nucleic acids as well as small nucleic acids, for example, by using triacrylate monomers instead of diacrylates [12, 47]. Moreover, due to their branched structure, they are also used for preparing hydrogels in cases of local delivery applications [48, 49].

**Table 1:** Summary of the main pBAE types, indicating their specific structure, the nucleic acid loaded, their intended application and the development stage

**pBAE subtypes**	**Chemical structure**	**Loading**	**Application**	**Important features for application**	**Development stage**	**Reference**
Bare pBAEs		pDNA; combination of pDNAs	Genetic diseases in the retina, fibroblasts reprograming; combined therapy hepatocellular carcinoma	First pBAEs described – cationic charge thatentraps the nucleic acids	In vivo mice models; in vitro; murine orthotopic models	[44–46]
Oligopeptide-end terminated		siRNA, mRNA	Breast cancer therapeutics, gene replacement of muscular dystrophies, gene edition of neurogastrointestinal encephalomyopathy and vaccination [39]	End oligopeptides decrease toxicity, thus allowing a higher dose administration.	Preclinical studies (mice)	[91]
Cytamine-terminated bioreducibled		siRNA coding for an inhibitor of osteo- genesis, B-cell lymphoma (Bcl)-like protein 2 (BCL2L2) [92]	Mesenchymal stem cells differentiation to promote osteogenesis	Bioreducible groups allow the triggered release of the nucleic acid only in certain conditions.	Only in vitro	[93]
Bioreducible linear pBAE		siRNA and mRNA	General, to encapsulate siRNA; and also antigenic mRNA	Same advantage as previous one.	Only polymer synthesis	[94]
Highly branched pBAE		Reporter plasmid DNA	Skin diseases	Having a higher cationic density, allow a higher encapsulation efficiency of the nucleic acid.	Preclinical in mice	[95]
Aliphatic heterocycle-containing pBAEs		NC1 control dicer-substrate siRNA (DsiRNA) dúplex (non-targeting nucleica cid)	To demonstrate the immunogenicity of the polymer itself	Thanks to their intrinsic immunogenicity, they can be used as autoadjuvants.	In vitro activation of macrophages	[65]
Hyperbranched PEG-modified pBAE		Reporter mRNA	Chronic wounds healing	They can be used for hydrogels formation, in addition to nanoparticles.	In vitro	[48]
Assymetric pBAEs for hydrogel formation		Plasmids	CRISPR/Cas gene editing	They allow the addition of different functionalities in a single polymer chain.	Preclinical (pigs)	[96]
Propionated pBAEs		Plasmid	Plasmid vaccination	More hydrophobic charges that provide stability.	In vitro	[67]
Other assymetric pBAEs		Plasmid + sgRNA	CRIPSR/Cas9 gene editing	Same advantages as other assymetric pBAEs.	In vitro	[97]

Monomers can also contain other functional groups in their lateral chains that are later used to functionalize the resulting particles. For example, in our group, we use the end-terminated acrylate pBAEs for attaching peptides via Michael’s addition of thiol from an N-terminal cysteine amino acid. Besides providing ionizable amine groups that increase polynucleotide loading capacity and transfection efficiency, these peptides can also increase the tissue specificity of the nanoparticles. Generally, we use cationic tripeptides (most commonly lysine, arginine, and histidine) for the end-caping, creating the so-called oligopeptide-terminated pBAEs (OM-pBAEs, Fig. 2C) [37–39]. Using cationic peptides facilitates the efficient electrostatic binding of the mRNA without synthetic, usually toxic cationic components. In addition, thanks to their pKa values close to organelles pH, tripeptide end-modification facilitates the exit of mRNA from the endosomes after cell uptake via the so-called proton sponge effect. Other examples include attaching a PEG chain to increase particle stability or reduce protein corona formation [50, 51]. High tissue affinity moieties such as sugars, small molecules, or peptides can also be attached to gain specificity in systemic administration [52–54].

## pBAEs for nucleic-acids delivery in inmunotherapies

### Advantages over competitors already in clinical practices

Targeting therapies specifically for modifying the immune system is full of intricacies. Specifically, eliciting unwanted immune system response can lead to fast nanoparticle clearance or even an immune disease. It is well known that many nanoparticle systems generate small unwanted humoral responses that might lead to therapy fail. For example, COVID vaccination based in Solid Lipid Nanoparticles (SLN), proven to be optimal for intramuscular administration in such infectious diseases as it elicited an immune system recruitment, similar to an adjuvant. This effect, however, is unwanted when using a systemic intravenous route for immunotherapies.

Indeed, despite the established efficacy of lipidic vectors, they are associated with the drawback of inducing undesired immune system responses. Additionally, the synthesis and further functionalization of lipidic vectors pose considerable challenges, limiting the incorporation of homing devices and resulting in off-target effects.

**Fig. 3:**
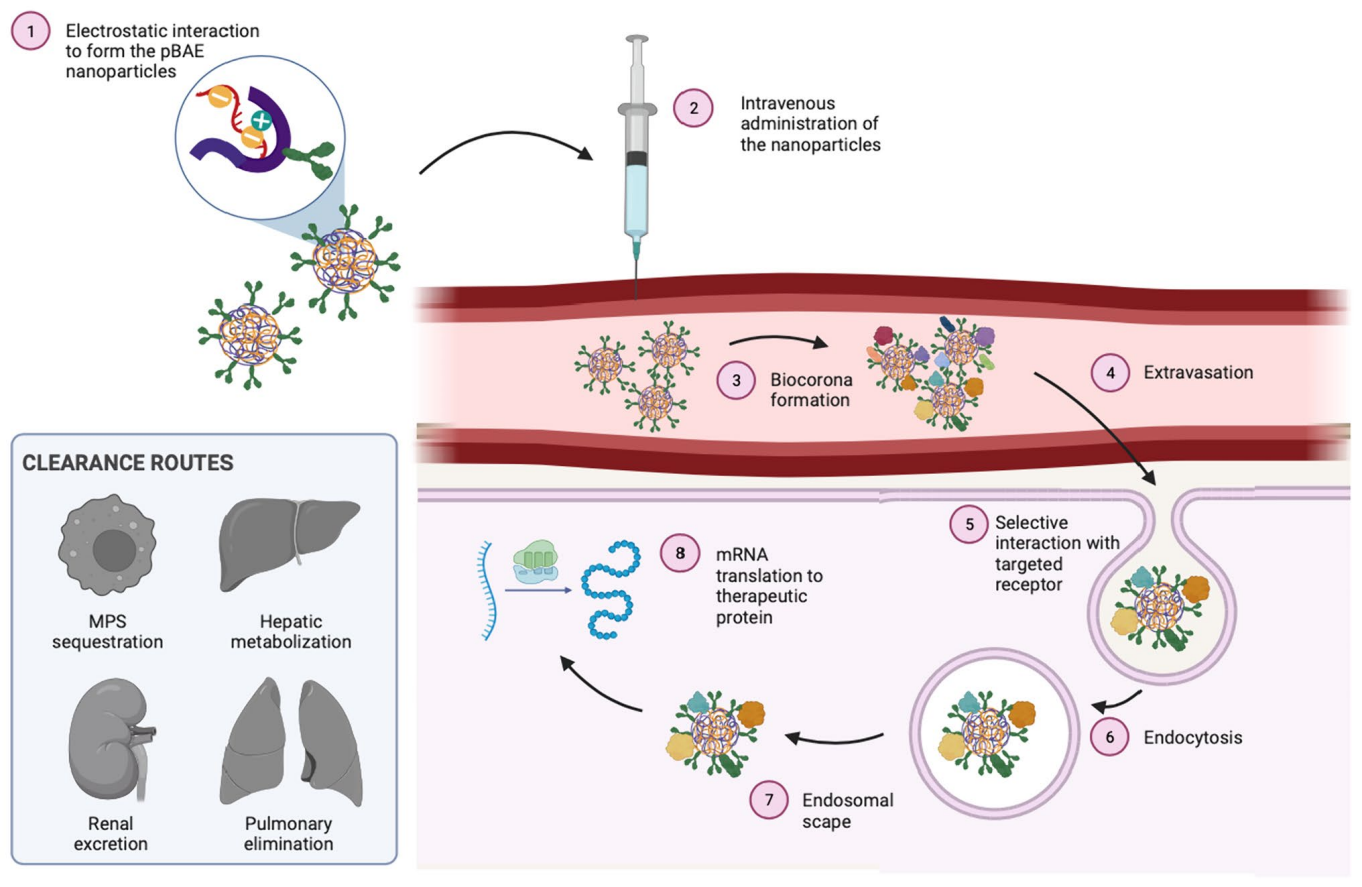
The journey of intravenously injected nanoparticles. Once systemically administered, pBAE nanoparticles will be embedded by blood proteins and other components, shifting their synthetic identity to their biological identity due to biocorona formation. Further, to reach their targets, nanoparticles will be extravased through transcytosis, thanks to their small nanometric size and, once in target tissues, they will interact with target receptors, to be then internalized in the cytoplasm. This process will take place through endocytosis, followed by the scape of the already decomplexed particles from the endosomes to the cytoplasm, where mRNA will be translated into protein. To note the need to eliminate already used polymer, which is done through degradative and excretion routes such as liver, urine or feces, as indicated in the left bottom part of the figure. These can also act as a premature clearance, if the nanoparticles are taken up by the mononuclear phagocyte system (MPS), but if well designed, they should avoid this premature clearance. to highlight that hepatic metabolization is the main degradative route for lipid nanoparticles, while polymeric nanoparticles (as pBAEs) are less eliminated by the liver. Also, the role of pulmonary clearance in the elimination of very big particles

Furthermore, the encapsulation efficiency of genetic cargo by positively charged lipids is generally low, requiring a high proportion of lipids relative to the genetic material. Genetic cargo is usually located at the periphery of these nanoparticles and exposed to nucleases, which is why they required extreme cold conditions for stability. In contrast, pBAE polymers offer a promising alternative by addressing these limitations, providing a more efficient and targeted approach to gene delivery with reduced risk of immune system reactions and enhanced encapsulation efficiency (Fig. 3).

Firstly, pBAEs are biocompatible and biodegradable, which avoids problems related to bioaccumulation and allows the direct in vivo use of high doses of the biotherapeutics. In a previous publication, we demonstrated the complete depletion of pBAE molecules 48 h after injection [55]. Body clearance was inferred due to the radioactive signal loss of the labelled pBAE polymers. Secondly, their synthesis is simple, rapid, and industrially scalable. Michael addition and ring-opening polymerization are examples of one-step economic synthesis that allow the polymer length to be controlled by stoichiometric adjustment. Moreover, their synthetic procedure allows for the tunability of pBAE structure to introduce structural diversity for the addition of different functionalities to, for example, increase hydrophobicity, tune the polymer architecture and molecular weigth [37–39]. Thirdly, and probably most importantly towards industrialization and commercialization, pBAE nanoparticles can be freeze-dried and redispersed without losing their properties. As demonstrated in earlier work, dry pBAE/polynucleotide nanoparticles can be stored for almost one year without losing functional capacity [56]. This is of utmost importance when considering the transference for clinical use since it facilitates logistics and storage costs while allowing the development of treatments for non-developed countries. Finally, producing the pBAE nanoparticles can be easily scalable under GMP conditions using microfluidics equipment [57].

### Possibility of direct in vivo use

Referring to their direct in vivo use, it is noteworthy to mention the capacity of pBAEs to efficiently encapsulate nucleic acids and protect them from nucleases in vivo. pBAEs allow the time and space control of their distribution after systemic administration. pBAE nanoparticle biodistribution is controlled by tuning their surface functionality (i.e., tethering PEG or peptide chains). Moreover, adding different ligand macromolecules during their synthesis allows for the selective targeting of target cells (see Fig. 2D for an extensive list of targeting moieties used). Targeting moieties can be covalently attached, or ligands can be included after their assembly by electrostatic or hydrophobic interactions [12]. In our own experience, hepatocytes can be targeted when covalently linking retinol. At the same time, its biodistribution is facilitated to the antigen-presenting cells by mannosylation, in both cases, using side-chains functionalization to preserve the cationic charge of the end-capping oligopeptides [58–60]. Green et al. [61] as a difference, coated polyplexes using polyglutamic acid (PGA) functionalized with the RGD peptide for endothelial cells targeting, as well as Smith et al. [62] who also used PGA for the T cells targeting through CD3 antibodies. These are only a few examples of the wide variety of (macro)molecules, mainly antibodies or their fragments, peptides, aptamers, and oligosaccharides that are reported to increase tissue specificity.

In addition to the ease of adding targeting moieties to their structure, pBAEs have another essential advantage when ensuring correct expression of the encapsulated genetic material, as they are endocytosed close to the plasma membrane. Once inside the endosomes, secondary and tertiary amines promote endosomal escape to the cytoplasm in a polymer pKa-controlled kinetics, thus allowing their use for nucleic acid delivery, which acts intracellularly [12, 22, 37, 38].

### Effects of biocorona in biological fluids

pBAE/polynucleotide nanoparticles active tissue targeting can be achieved in controlled experiments; however, when in contact with complex biological media (i.e., plasma proteins), this specificity is, in most cases, disappeared. Indeed, as the vectors tend to have a slightly positive surface charge when in contact with serum proteins, these uncontrollably adsorb on the particle’s surface and bury all binding motifs. At this point, the effect that this so-called corona has on modifying any nanoparticle surface is noteworthy. Once synthesized, the nanoparticles’ surface is defined by their synthetic identity; this is the chemical groups of the biomaterials that are exposed on the nanoparticle surface, where a targeting moiety is commonly added to direct the nanoparticles selectively to the cells of interest. Nevertheless, when nanoparticles enter in contact with physiological fluids, the biocorona transforms their surface in their biological identity, meaning that their surface is modified due to the attachment of proteins and other macromolecules from blood and other biological fluids, thus hiding important surface functionalities and hampering their expected biodistribution [12, 63]. Moreover, the adsorption of opsonins or other specific proteins might lead to fast clearance from the body. pBAE nanoparticles, as most nanocarriers, include hydrophilic polymers to overcome this common bottleneck step. Poly (ethylene glycol) (PEG) is the most extensively used polymer in drug delivery to allow the vectors to circumnavigate biological barriers, decrease the unspecific absorption in non-targeted tissue, and increase the specific cellular uptake [12]. However, PEG can be recognized by the immune system and causes strong allergic reactions in some patients [39, 40]. Consequently, extensive work on controlling the formation of the biocorona by tuning the nanoparticle’s surface exists in the bibliography. In our first attempts, we worked on tuning nanoparticle surfaces to promote the binding of proteins of interest from the corona. We published this study in 20196, selecting liver targeting as a proof-of-concept. Being aware that hepatocytes have receptors for the retinol-binding protein (RBD), a protein present in the human serum, we designed a retinol-modified place intending to promote the formation of a corona enriched with RBP. This work demonstrated that we could tune the biodistribution by playing with the protein corona while achieving the envisaged organ targeting. Nevertheless, this strategy is limited to targeting cells with specific affinity proteins in the serum. More work is needed to achieve satisfactory control of protein corona formation on pBAE nanoparticles, namely by designing new polymer or surface masks for achieving optimal biodistribution and therapy efficacy.

Thanks to all these advantages, although not yet in clinics, the number of studies reporting different variants of pBAEs for nucleic acid encapsulation and treatment of many unmet medical needs is enormous. In addition to our group, the work of Jordan J. Green and the collaborators’ group using pBAEs is also remarkable. Table 1 shows a schematic summary of the main pBAE studies classified as a function of the specific pBAE structure, indicating their intended application.

## The good: use for cancer immunotherapy

**Fig. 4:**
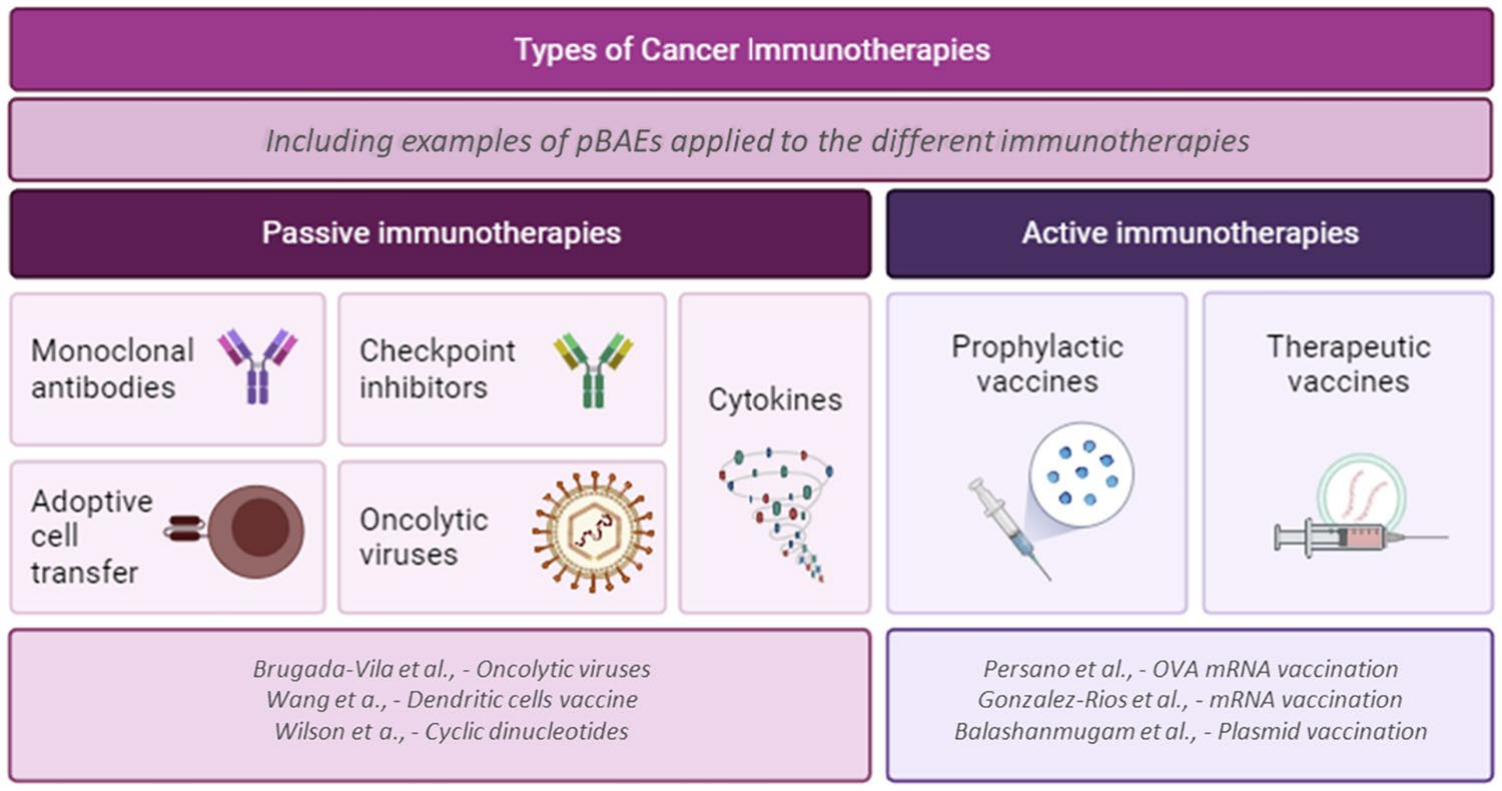
Scheme of cancer immunotherapy types, including types, examples and references that use pBAEs

Cancer immunotherapy refers to any therapeutic strategy devoted to (re) activating the immune system to recognize and eliminate tumor cells (see Fig. 4 for immunotherapy types). Beyond chimeric antigen receptor (CAR)-T cell therapies for hematological malignancies and the vastly used ICIs, primarily to treat solid tumors, cancer vaccination stands as the promise of the near future curation of cancer [2, 64].

pBAE polymers, given their robustness in formulating nanoparticles with different loadings, physicochemical properties, and interactions with the body, have been extensively studied as the ideal candidates to boost immunotherapies to clinics, from innate immune stimulation, cancer vaccination, adoptive cell therapies, and oncolytic virotherapy [12, 20, 22, 39, 65–69].

This section details some examples of the most representative studies reporting the use of pBAEs for cancer immunotherapy. Our aim was not to extensively revise all available literature on the field but to highlight the most important results in the field. The interested reader is referred to excellent reviews on the fundamentals for immunotherapy and a deep discussion of potential therapies, such as those from Zhang, Liu, Cutlar and Karlsson, for example [12, 26, 47, 70].

### Cancer vaccination

Cancer vaccination involves medical interventions to prevent or treat cancer by mobilizing the adaptive immune response against tumor antigens. While traditional vaccines traditionally consisted of attenuated or inactivated viruses, applying viral vectors to cancer, excluding those linked to viral infections, becomes challenging due to issues with antigen selection. Defining tumor antigens presents a formidable challenge, with neoantigens—those exclusive to tumors—being the preferred option. For practical purposes, selecting tumor-associated antigens is often necessary to avoid the excessive personalization of therapies [2, 64, 71].

Using antigens as mRNA macromolecules in non-viral vectors could be advantageous in this context. Since mRNA macromolecule structure is quite similar independently to the codified antigen, they are preferred to protein/peptide antigens, which would require the re-formulation of the vaccine each time the antigen is changed. Additionally, mRNA’s intrinsic immunogenicity does not require the addition of adjuvants, which is commonly controversial in traditional vaccine formulations [72, 73]. Finally, mRNA is directly translated to proteins in the cytoplasm.

Specifically for the design of mRNA vaccines, cationic polymers could become the game changer technology, and pBAEs have already been used for this purpose. Combining with a lipid-coating, Persano et al. [74] demonstrated that ovalbumin (OVA) mRNA subcutaneous vaccination immunized mice, as confirmed by an increase in interferon (IFN)-b levels in serum, activation of T and other immune cells in lymph nodes and antigen-specific melanoma cells killing. Accordingly, Ben-Akiva et al. [68] used lipid-modified bioreducible pBAEs to co-delivery antigenic mRNA with toll-like receptor adjuvants, demonstrating the efficacy of these nanoparticles to elicit an antitumor immune response in mice models of colon adenocarcinoma and melanoma. In our hands, OVA mRNA vaccination was also demonstrated to have a melanoma therapeutic effect and a more impressive prophylactic effect in mice models. When we subcutaneously treated healthy mice with our pBAE nanoparticles, targeted to the Clec-9 A receptor, overexpressed in dendritic cells, the tumor could not even be implanted, thus avoiding not only the formation of metastasis but also the primary tumor (patent accepted [75] and results submitted for publication).

The number of cancer vaccination studies using DNA is considerably lower compared of those of mRNA. The reason behind this striking outcome is that DNA subcellular localization might lead to cell genome integration and consequently possess an oncogenic potential. Despite these possible outcomes, the pBAE/DNA nanoparticles can also be prepared using the same principles used for RNA. Despite this, pBAEs loaded with DNA are generally bigger in size (in our own experience) and depending on the gene size, encapsulation might be challenging. To overcome this issue, Balashanmugam et al. [67] developed pH-sensitive pBAE/(lactic-co-glycolic) (PLGA) polymer microparticles for the encapsulation of giant DNA, thus seeding the first steps of DNA vaccination.

Adoptive cell transfer (ACT) therapies, the most widely developed CAR-T cell therapies, can be achieved using pBAEs as non-viral gene delivery vehicles. Kim et al. [50], have extensively worked on in vivo targeting T cells with leukemia-specific CAR DNA-loaded pBAE nanoparticles. They used CD3-antibody surface-modified PGA/pBAE nanoparticles, including nuclear localization signals in the polymer, to achieve the transfection of circulating T cells, demonstrating that the use of a DNA encoding for the CAR receptor was able to selectively transfect them, thus allowing a long-term remission of leukemia in a mice model of B-cell acute lymphoblastic leukemia (ALL) [6]. They went a step further beyond ACT, demonstrating that T cells in vivo targeting and transfection using selective pBAEs was also helpful for the expression of mRNAs that knocked down anti-cancer genes of T cells, expressed transcription factors of memory formation in T cells and reprogram self-renewal properties typical from hematopoietic cells.

Referring to immune therapies based on immune checkpoint inhibitors or ICIs (i.e., antibodies against the molecular triggers of immune cell activation), they have only been used as combined therapies, along with pBAE nanoparticles, as in the example of Zou et al., who administered anti-PDL1 antibodies in combination with mannosylated pBAE nanoparticles, used as a melanoma vaccine [76]. Encapsulation of these antibodies in pBAE nanoparticles is not expected to provide any advantage since, being antibodies, they are stable in physiological fluids and naturally targeted to the immune checkpoints of interest with a high affinity.

Another essential issue to consider when designing cancer immunotherapies is the common immune suppression found in tumor microenvironment (TME). Different immune system mechanisms, such as macrophage polarization and immune checkpoint inhibitors overexpression, could be modulated to revert it. To tackle these mechanisms, Dold et al. [65] deepened into a mechanistic study of the pBAE effects regarding the immune microenvironment in tumors. They found that, although pBAEs can activate antigen-presenting cells (APCs), as we demonstrated previously [60], this activation is independent of the immune stimulatory receptors of the family of toll-like receptors (TLR) and NfkB signaling pathways. Still, they activate interferon (IRF), an important inflammatory pathway, an important finding to allow a more rational design of immunotherapeutic nanoparticles. Also, immune modulation to revert immune suppression in the TME can be achieved by delivering immune modulator molecules. Wilson et al. [22] did this in their study by loading cyclic dinucleotides (CDN) in pBAE nanoparticles as adjuvants to trigger an antitumor immune response. Although they achieved higher cytosolic accumulation of the CDNs compared to the naked nucleotides, a very high polymer: nucleic acid ratio was required to encapsulate these short nucleic acids efficiently.

**Fig. 5:**
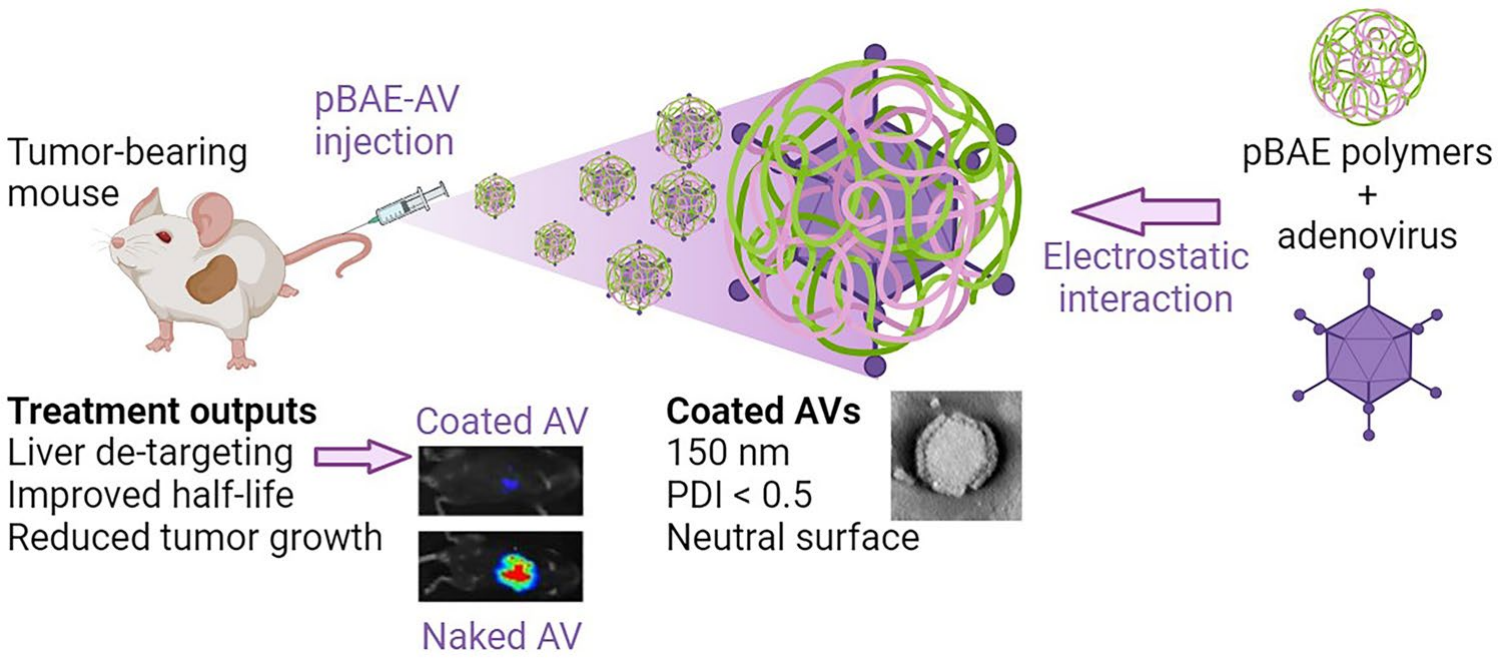
Schematic representation of adenoviruses coating by pBAEs. Results are summarized from ourselves results published in Brugada-vilà et al. [66]

All the examples here refer to using pBAEs to formulate non-viral delivery systems. However, hybrid polymer/viral vector-based strategies have also been developed for cancer vaccination and other existing immunotherapies [77, 78]. Viral-based therapies are often limited by pre-existing anti-vector immunity, which may decrease the treatment efficacy. pBAEs have also been used to circumvent immune system viral neutralizing antibodies. For example, in our group, we demonstrated pBAEs applicability to improve the performance of adenoviruses [66]. Tacking pancreatic cancer as proof-of-concept disease, we coated adenoviral vectors to avoid their premature physiological clearance caused by the high seroprevalence of anti-adenovirus neutralizing antibodies in most humans. Using a pBAE-coated oncolytic adenovirus, we showed how the coating enabled a five-fold increase of the virus circulating time in blood, thus allowing to boost the antitumor efficacy in a model of pancreatic ductal adenocarcinoma mice model, as compared to the naked virus (Fig. 5 with summarized results).

## The bad: current challenges of pBAEs for cancer immunotherapy

Despite the significant advantages that pBAEs possess compared to other cationic polymers, they still have some issues to overcome. These are mainly related to possible cytotoxicity, chemical and colloidal stability, and GMP production. Combining these reasons has halted the disembarking into clinics and remains a vigorous research topic.

### Possible toxicities

First, it must consider the possible immunogenicity of the pBAE polymers by themselves [12]. Immunogenicity presents challenges in autoimmune disease therapies, but in the context of cancer immunotherapy, the inherent immunogenicity of biomaterials holds the potential to be advantageous. Nevertheless, uncontrolled immunogenicity raises concerns, as previously explored by Dold et al. [65] who conducted a review of various pBAE structures that exhibited specific pro-inflammatory signals [54, 71, 79]. In fact, we also found coherent results in some of our studies, where pBAE polymers produced a non-depreciable pro-inflammatory cytokines response, attributed to the neat polymers. After nanoparticle surface modification, this response disappeared (results submitted for publication). As they stated, even the intrinsic immunogenicity of the pBAEs as biomaterials for tumor immunotherapeutic purposes, it is of crucial importance to recognize the mechanism by which this inflammation is driven to allow a more rational design of pBAE polymers depending on the intended therapeutic use.

Also related to eventual cytotoxicity, the fact that pBAE nanoparticles’ surface charge is cationic makes them interact with all plasmatic membranes, which, in addition to hampering selectivity, as mentioned above, could disrupt cell membranes. This is a known and solved problem, as reported in previous studies, such as Fields et al., who coated the particles with a PLGA and modified them with cell-penetrating peptide (CPP). We observed the same effect by coating the nanoparticles with polysaccharides (i.e., chitosan) to avoid the excessive cationic charge and reduce its interaction with lung mucosa [12, 29, 48, 77–82].

### Stability matters

pBAEs robustness is a fact supported by numerous studies reporting the encapsulation of various nucleic acid types. Compared to other cationic polymers, it must be considered that, as compared to other cationic polymers, pBAEs cationic charge density is lower, and commonly, high ratios are required for efficient nucleic acid encapsulation [12]. In terms of encapsulation efficiency, either DNA or RNA can be encapsulated [39, 40, 60, 82]. Nevertheless, the encapsulation efficiency of oligonucleotides, such as small RNAs or CpG stimulator of interferon genes (STING) agonists, widely used as boosters of innate immunity, is decreased, thus requiring a notably higher proportion of the polymer or resulting in polyplexes that may lose their stability easily [83–85]. We have also observed in the case of coating adeno-associated viral vectors [86]. Solely using electrostatic interactions did not result in a solid attachment to the virus surface, which we attributed to the lower surface charge density of the adeno-associated capsid. Thus, the covalent union between the polymer and the cargo is likely necessary in some cases, posing other problems, such as the need for a reversible interaction to release the nucleic acid.

Making use of electrostatic interaction may also become a problem for the use of non-invasive routes of administration. The advantageous fact of the facilitated release of the functional nucleic acid thanks to the reversibility of the electrostatic interaction is, at the same time, an issue when it comes to low invasive routes of administration, such as mucosa, where the presence of salts and oligosaccharides usually competes with nucleic acids and polymers, thus disassembling nanoparticle’s structure. Additionally, the cationic surface charge of pBAE nanoparticles makes them highly promiscuous, tending to interact with any cell membrane, all composed of anionic phospholipid bilayers. Again, this is an advantageous property when aiming to achieve a high transfection, which becomes an issue when trying to be selective and could bring associated intrinsic toxicity depending on the cationic chemical group used [12]. From this point of view, using non-invasive administration routes, such as the inhaled or the subcutaneous, is limited by the maximum injection volume. Since pBAE nanoparticles may have solubility issues in physiological fluids, arriving at therapeutic doses could also become a problem [12]. Consequently, the covalent binding could again be an alternative in terms of enabling efficient encapsulation of short RNAs without the need for high ratios of polymer and stabilizing the nanoparticles in physiological fluids.

### Path through clinics

Another challenge of pBAEs is the lack of efficient clinical-grade product preparation methods. First, the purification methods could be challenging both from the point of view of the polymers but also the perspective of the final nanoparticle formulation. Because pBAE polyplexes rely on electrostatic interactions, complete encapsulation is not always possible, leaving polymers or polynucleotides free. In principle, since naked nucleic acids cannot penetrate cells, nevertheless, taken into account the immunogenic character of some of them, they could trigger an immune system rejection. Moreover, we previously reported the presence of dissolved polymer molecules in nanoparticle formulations [82, 87]. Although detrimental in regulatory issues, these non-complexed molecules were necessary to allow the functionality of the polyplexes since their removal dramatically decreased the transfection efficiency of the particles. Thus, we hypothesized that polymer molecules are in a constant dynamic (macro)molecular interchange, eventually making them part of the nanoparticle.

Finally, the preparation method of nanoparticles by manual pipetting is required, making scaling up difficult. This issue could be solved using microfluidic devices, which, in addition to automatization, allow for scaled-up under good manufacturing practices (GMP) production conditions. There are only few articles demonstrating the use of microfluidics for pBAE formulation [81, 85]. The most common microfluidic approach for producing polymeric NPs is hydrodynamic flow focusing (HFF), using either 2D or 3D models. Each HFF model has its own unique characteristics and applicability more research is still required to confirm the change in the preparation method does not change the pBAE nanoparticle formulations [88, 89].

## The future: what is missing in the path towards clinics?

pBAEs bring numerous advantages and specific properties that make them attractive candidates for their use in cancer immunotherapies. However, critical stepping-stones do not allow them their transition from bench to clinic. Critically speaking, these challenges are related to regulatory concerns, logistics that might arise during industrialization and managing and designing more efficient immunotherapies from the (bio)molecular point of view.

### Regulatory issues

From the regulatory point of view, not having any commercial product composed of pBAEs requires a detailed study of these polymers’ safety in humans, the biomaterial itself, and the whole nano-system. At this point, it is noteworthy that most successful pBAE nanoparticles comprise more than one polymer variant, meaning that the regulatory approval becomes intricate. The need for the combination of two or more polymer variants in the same nanoparticle is a double-edged sword. It is advantageous in providing the particles with more than one functionality [38, 40]. In one of our recent publications, where pBAEs were used for tracking purposes, we were required to combine the so-called C6 pBAE, incorporating a hydrophobic lateral chain, with the so-called C32 polymer, having the lateral chains terminated with an alcohol group, required for their further functionalization with the radiolabeled ammonium trifluoroborate residue [55]. Also, when incorporating active targeting moieties, the need for only a fraction of the polymer, including the targeting to avoid the saturation of the cell surface receptors, makes the use of a polymer combination unavoidable. This happened, for example, in our recent research, where we added mannose groups in the lateral chains of the pBAE for the antigen-presenting cells targeting. We demonstrated the importance of a low fraction of the polymer to be functionalized [60]. Thus, the system’s complexity is a factor that makes the path through clinics slower.

Also, related to regulatory approval, there is the need for scaled-up and under suitable manufacturing protocols (GMP) conditions of production. As discussed in the previous section, even though theoretically, it is feasible to produce at a large scale, no public studies report it. Being optimistic, since the number of pBAE patents is large (see Table 2 with the most relevant summarized), we hypothesize that, as in our self-situation, there are commercial interests behind that make that, although the scaling-up protocols have already been started, no public information is available yet.

**Table 2:** Relevant examples of patents involving pBAEs polymer variants (2020–2023)

**Patent title**	**Year of publication**	**What protects**	**Number**	**Reference**
Bioreducible Poly (Beta-Amino Ester)s For siRNA Delivery	2019	Degradable polymers were synthesized that self-assemble with nucleic acids, proteins, hydrophobic drugs, and other small molecules to form particles that are effective for delivery into a cell, tissue and/or organism either in vitro or in vivo.	US20190209690A9	[98]
Polymer-Encapsulated Viral Vectors for In Vivo Genetic Therapy	2020	Polymer-encapsulated viral vector nanoparticles and methods of using them provide enhanced delivery of genetic material for use in gene therapy and other applications.	WO2019145796A2	[99]
Targeted poly(beta-amino esters)	2021	Non-toxic targeted poly beta-amino esters (PBAEs) are synthesized by using click chemistry to attach a targeting moiety.	WO2021191686A1.	[100]
Peptide modulators of angiogenesis and uses thereof	2021	Polymeric nanoparticles, microparticles, and gels for delivering cargo, e.g., a therapeutic agent, such as a peptide, to a target, e.g., a cell, and their use for treating diseases, including angiogenesis-dependent diseases, such as age-related macular degeneration and cancer, are disclosed.	US20210220287A1	[101]
Highly stable biodegradable gene vector platforms for overcoming biological barriers	2021	Colloidally stable non-viral gene vector delivery systems capable of overcoming various biological barriers, are disclosed. Methods of formulating the vectors are also provided.	US11007279B2	[102]
Zwitterionic functionalized poly(beta-aminoester) polymers and uses thereof	2021	The invention provides a poly(beta-aminoester) polymer of formula or a pharmaceutically salt thereof which comprises one or more zwitterionic polymers.	EP4166594A1	[103]
Core/shell structure platform for immunotherapy	2022	Biocompatible core/shell compositions suitable for the delivery of populations of mRNA molecules to mammalian cells.	US20220305103A1	[104]
Poly(beta-amino ester)-co-polyethylene glycol (peg-pbae-peg) polymers for gene and drug delivery	2023	Polyethylene glycol (PEG)-b-poly(β-amino ester) (PBAE) co-polymers (PEG-PBAE) and blends of PEG-PBAEs and PBAEs and their use for delivering drugs, genes, and other pharmaceutical or therapeutic agents.	US20230107757A1	[105]
Polymeric nanoparticle genetic vaccines	2023	Compositions comprising degradable polymers combined with nucleic acids, such as DNA and RNA, encoding antigen and their use as genetic vaccines are disclosed.	WO2023056293A1	[106]
Covalently coated adeno-associated virus vector for its use in gene therapy	2023	It is provided an adeno-associated virus (AAV) vector particle, wherein the AAV has at least one capsid protein having one N-terminal covalently bond to a poly(beta aminoester) (PBAE), or a pharmaceutically acceptable salt thereof.	WO2023006651A1	[107]

### Logistic issues

Another issue to consider when thinking about clinical translation is the logistics of storage, transportation, distribution, and administration of the medicines. Nanomedicine formulations are commonly in liquid form. These liquid formulations are much less stable than solid ones. As a nucleic acid’s active principle, its stability is compromised at room or even mild cold temperatures. Thus, extreme cold refrigeration is required for genetic cargo. This need was already observed in COVID-19 vaccines, which are supposed to be a challenge in terms of the availability of freezers. Consequently, some studies [44], including ours, started to set up protocols for freeze-drying the formulations, which is commonly a bottleneck step in nanomedicine. This is advantageous in terms of stability, as formulations only require a dry environment and not extremely cold freezers, thus facilitating storage and distribution costs while allowing their use in regions where this specialized equipment is unavailable. However, this could represent an issue for the administration. Since the formulation needs to be redispersed in a specific volume, certain local administration routes may not reach therapeutic doses, allowing only a significantly reduced volume.

### Complex diseases: how to handle them?

Many unmet medical needs are complex diseases, such as cancer, with more than one metabolic route affected. Thus, a combination therapy, including more than one active principle, may be required to revert their pathogenicity. Again, the formulation gets more convoluted, and, in addition to the complications of the regulatory procedure, it involves a preliminary stage of designing an optimized formula that allows for the efficient encapsulation of all the components together. The complexity intensifies when considering the diverse release kinetics associated with distinct active principles. For instance, mRNAs reach their maximum expression in vitro within 24 h, whereas microRNAs necessitate over 72 h to become active. Compounding this challenge is the need to target different cells within the intricate tumor microenvironment. For instance, siRNAs designed to silence oncogenes must be directed at tumor cells, while antigenic mRNAs should specifically target dendritic cells. Although addressing this issue remains unresolved, ongoing research exploring non-canonical modifications of mRNA for regulating transient expression in targeted cells holds promise in alleviating this drawback [90].

## Conclusions

Genetic-based immunotherapies hold great potential for treating various unmet medical needs, including infectious diseases, oncology, and metabolic disorders. During the last decade, we have reached a level of molecular understanding of the processes that lead to pathogenesis, especially in the area of cancer. In stark contrast, the advances in delivery vectors have been masked by several issues, including their chemical/colloidal stability, specificity, and (bio)compatibility). Designing new, efficient, and (bio)safe delivery vectors is critical to the therapy’s in vivo safety and efficacy. pBAEs have been designed and successfully used for nucleic acid delivery in cancer immunotherapy for the last fifteen years. Their intrinsic properties: biocompatibility, biodegradability, a cationic charge that allows electrostatic complexation of the nucleic acids, robustness, versatility, and tunability in design, make them ideal candidates for the in vitro, ex vivo, and in vivo delivery of a wide variety of nucleic acid types, from micro RNAs to long plasmids. Notably, the possibility of including surface functionalization has allowed essential milestones, from the possibility of achieving freeze-dried formulations to the selective in vivo transfection of only targeted cell subsets. Besides commercial interest, reaching a clinical application will require more research into pBAEs industrialization capacity, long-term stability, and logistics. In this sense, more research into the applicability of microfluidics for the large-scale production of pBAE nanoparticles is key. Moreover, specifically for cancer immunotherapeutics, the correct selection of tumor-specific antigens is also a critical parameter to go in hand when designing new delivery systems. Finally, a better molecular understanding of the intricate interaction between pBAE nanoparticles and complex biological media will allow for the design of new and more efficient delivery vehicles.

## Data Availability

The datasets generated during and/or analyzed during the current study are available from the corresponding author on reasonable request.

## References

[1] Faget J, et al. Neutrophils and snail orchestrate the establishment of a Pro-tumor Microenvironment in Lung Cancer. Cell Rep. 2017;21:3190–204. 29241546 10.1016/j.celrep.2017.11.052

[2] García-Fernández C, Fornaguera C, Borrós S. Nanomedicine in non-small cell lung cancer: from conventional treatments to immunotherapy. Cancers (Basel). 2020;12:1–26. 10.3390/cancers12061609 PMC7352459 32570729

[3] Hanna NH, et al. Therapy for stage IV non-small-cell lung cancer with driver alterations: ASCO and OH (CCO) joint guideline update. J Clin Oncol. 2021;39:1040–91. 33591844 10.1200/JCO.20.03570

[4] Hanna NH, et al. Therapy for stage IV non–small-cell lung cancer without driver alterations: ASCO and OH (CCO) joint guideline update. J Clin Oncol. 2020;38:1608–32. 31990617 10.1200/JCO.19.03022

[5] Walsh RJ, Soo RA. Resistance to immune checkpoint inhibitors in non-small cell lung cancer: biomarkers and therapeutic strategies. Ther Adv Med Oncol. 2020;12:1–22. 10.1177/1758835920937902 PMC7339077 32670423

[6] Pérez-Herrero E, Lanier OL, Krishnan N, D’Andrea A, Peppas NA. Drug delivery methods for cancer immunotherapy. Drug Deliv Transl Res. 2023. 10.1007/s13346-023-01405-9. 37587290 10.1007/s13346-023-01405-9 PMC10746770

[7] Barenholz Y. Doxil® - the first FDA-approved nano-drug: lessons learned. J Controlled Release. 2012;160:117–34. 10.1016/j.jconrel.2012.03.020 22484195

[8] Dilliard SA, Siegwart DJ. Passive, active and endogenous organ-targeted lipid and polymer nanoparticles for delivery of genetic drugs. Nat Rev Mater. 2023;8:282–300. 10.1038/s41578-022-00529-7. 36691401 10.1038/s41578-022-00529-7 PMC9850348

[9] Fornaguera C, et al. PLGA nanoparticles prepared by nano-emulsion templating using low-energy methods as efficient nanocarriers for drug delivery across the blood-brain barrier. J Control Release. 2011;211:134–43. 10.1016/j.jconrel.2015.06.002 26057857

[10] Maksimenko O, et al. Doxorubicin-loaded PLGA nanoparticles for the chemotherapy of glioblastoma: towards the pharmaceutical development. Int J Pharm. 2019;572:118733. 31689481 10.1016/j.ijpharm.2019.118733

[11] Chen Y, et al. Enzymatic PEGylated poly(lactone-co-β-amino ester) nanoparticles as biodegradable, biocompatible and stable vectors for Gene Delivery. ACS Appl Mater Interfaces. 2016;8:490–501. 26673948 10.1021/acsami.5b09437

[12] Karlsson J, Rhodes KR, Green JJ, Tzeng SY. Poly(beta-amino ester)s as gene delivery vehicles: challenges and opportunities. Expert Opin Drug Deliv. 2020. 10.1080/17425247.2020.1796628. 32700581 10.1080/17425247.2020.1796628 PMC7658038

[13] Perni S, Prokopovich P. Poly-beta-amino-esters nano-vehicles based drug delivery system for cartilage. Nanomedicine. 2017;13:539–48. 27746232 10.1016/j.nano.2016.10.001 PMC5339075

[14] Cordeiro RA, Serra A, Coelho JFJ, Faneca H. Poly(β-amino ester)-based gene delivery systems: from discovery to therapeutic applications. J Control Release. 2019;310:155–87. 31454533 10.1016/j.jconrel.2019.08.024

[15] Paunovska K, Loughrey D, Dahlman JE. Drug delivery systems for RNA therapeutics. Nat Rev Genet. 2022;23:265–80. 10.1038/s41576-021-00439-4. 34983972 10.1038/s41576-021-00439-4 PMC8724758

[16] Parés M, et al. Preclinical assessment of a gene-editing approach in a mouse model of mitochondrial neurogastrointestinal encephalomyopathy. Hum Gene Ther. 2021;32:1210–23. 34498979 10.1089/hum.2021.152

[17] Lynn DM, Langer R. Degradable poly(β-amino esters): synthesis, characterization, and self-assembly with plasmid DNA. J Am Chem Soc. 2000;122:10761–8.

[18] Moghimi SM, Hunter AC, Murray JC. Long-circulating and target-specific nanoparticles : theory to practice. Pharmacol Rev. 2001;53:283–318. 11356986

[19] Shi D, et al. To PEGylate or not to PEGylate: immunological properties of nanomedicine’s most popular component, polyethylene glycol and its alternatives. Adv Drug Deliv Rev. 2022;180:114079. 34902516 10.1016/j.addr.2021.114079 PMC8899923

[20] Kozielski KL, Rui Y, Green JJ. Non-viral nucleic acid containing nanoparticles as cancer therapeutics. Expert Opin Drug Deliv. 2016;13:1475–87. 27248202 10.1080/17425247.2016.1190707 PMC5021566

[21] Kozielski KL, Tzeng SY, De Mendoza H, Green JJ. Bioreducible cationic polymer-based nanoparticles for efficient and environmentally triggered cytoplasmic siRNA delivery to primary human brain cancer cells. ACS Nano. 2014;8:3232–41. 24673565 10.1021/nn500704t PMC4004313

[22] Wilson DR, et al. Biodegradable STING agonist nanoparticles for enhanced cancer immunotherapy. Nanomedicine. 2017;17:30193–4. 10.1016/j.nano.2017.10.013 PMC6035751 29127039

[23] Kamat CD, et al. Poly(β-amino ester) nanoparticle delivery of TP53 has activity against small cell lung cancer in vitro and in vivo. Mol Cancer Ther. 2013;12:405–15. 23364678 10.1158/1535-7163.MCT-12-0956 PMC3624031

[24] Guerrero-Cázares H, et al. Biodegradable polymeric nanoparticles show high efficacy and specificity at DNA delivery to human glioblastoma in vitro and in vivo. ACS Nano. 2014;8:5141–53. 24766032 10.1021/nn501197v PMC4046784

[25] Tzeng SY, et al. Non-viral gene delivery nanoparticles based on poly(β-amino esters) for treatment of glioblastoma. Biomaterials. 2011;32:5402–10. 21536325 10.1016/j.biomaterials.2011.04.016 PMC3118545

[26] Zhang J, et al. Poly(β-amino ester)s-based nanovehicles: Structural regulation and gene delivery. Mol Ther Nucleic Acids. 2023;32:568–81. 10.1016/j.omtn.2023.04.019. 37200860 10.1016/j.omtn.2023.04.019 PMC10185705

[27] Magana JR, Sproncken CCM, Voets IK. On complex coacervate core micelles: structure-function perspectives. Polym (Basel). 2020;12:1953. 10.3390/polym12091953 PMC7565781 32872312

[28] Cabral H, Miyata K, Osada K, Kataoka K. Block copolymer micelles in nanomedicine applications. Chem Rev. 2018;118:6844–92. 29957926 10.1021/acs.chemrev.8b00199

[29] Fischer D, Li Y, Ahlemeyer B, Krieglstein J, Kissel T. In vitro cytotoxicity testing of polycations: influence of polymer structure on cell viability and hemolysis. Biomaterials. 2003;24:1121–31. 12527253 10.1016/s0142-9612(02)00445-3

[30] Wagner M, Rinkenauer AC, Schallon A, Schubert US. Opposites attract: influence of the molar mass of branched poly(ethylene imine) on biophysical characteristics of siRNA-based polyplexese. RSC Adv. 2013;3:12774.

[31] Cai J, et al. Effect of chain length on cytotoxicity and endocytosis of cationic polymers. Macromolecules. 2011;44:2050–7.

[32] Fichter KM, Ingle NP, McLendon PM, Reineke TM. Polymeric nucleic acid vehicles exploit active interorganelle trafficking mechanisms. ACS Nano. 2013;7:347–64. 23234474 10.1021/nn304218q PMC3586558

[33] Taori VP, Lu H, Reineke TM. Structure–activity examination of poly(glycoamidoguanidine)s: glycopolycations containing guanidine units for nucleic acid delivery. Biomacromolecules. 2011;12:2055–63. 21506608 10.1021/bm101537f

[34] Van Bruggen C, Hexum JK, Tan Z, Dalal RJ, Reineke TM. Nonviral gene delivery with cationic glycopolymers. Acc Chem Res. 2019;52:1347–58. 30993967 10.1021/acs.accounts.8b00665

[35] Liu Y, Reineke TM. Degradation of poly(glycoamidoamine) DNA delivery vehicles: polyamide hydrolysis at physiological conditions promotes DNA release. Biomacromolecules. 2010;11:316–25. 20058913 10.1021/bm9008233

[36] Little SR, et al. Poly-β amino ester-containing microparticles enhance the activity of nonviral genetic vaccines. Proc Natl Acad Sci USA. 2004;101(26):9534–9. 15210954 10.1073/pnas.0403549101 PMC470709

[37] Segovia N, Dosta P, Cascante A, Ramos V, Borrós S. Oligopeptide-terminated poly(β-amino ester)s for highly efficient gene delivery and intracellular localization. Acta Biomater. 2014;10:2147–58. 24406199 10.1016/j.actbio.2013.12.054

[38] Dosta P, Segovia N, Cascante A, Ramos V, Borrós S. Surface charge tunability as a powerful strategy to control electrostatic interaction for high efficiency silencing, using tailored oligopeptide-modified poly(beta-amino ester)s (pBAEs). Acta Biomater. 2015;20:82–93. 25839122 10.1016/j.actbio.2015.03.029

[39] Fornaguera C, et al. mRNA delivery system for targeting antigen-presenting cells in vivo. Adv Healthc Mater. 2018. 10.1002/adhm.201800335. 29923337 10.1002/adhm.201800335

[40] Navalón-López M, Dols-Perez A, Grijalvo S, Fornaguera C, Borrós S. Unravelling the role of individual components in pBAE/polynucleotide polyplexes in the synthesis of tailored carriers for specific applications: on the road to rational formulations. Nanoscale Adv. 2023. 10.1039/d2na00800a. 36926558 10.1039/d2na00800a PMC10012844

[41] Sunshine JC, Akanda MI, Li D, Kozielski KL, Green JJ. Effects of base polymer hydrophobicity and end-group modification on polymeric gene delivery. Biomacromolecules. 2011;12:3592–600. 21888340 10.1021/bm200807s PMC3959121

[42] Akinc A, Anderson DG, Lynn DM, Langer R. Synthesis of poly(β-amino ester)s optimized for highly effective gene delivery. Bioconjug Chem. 2003;14:979–88. 13129402 10.1021/bc034067y

[43] Zugates GT, et al. Gene Delivery properties of End-modified poly(β-amino ester)s. Bioconjug Chem. 2007;18:1887–96. 17929884 10.1021/bc7002082

[44] Sunshine JC, Sunshine SB, Bhutto I, Handa JT, Green JJ. Poly(β-amino ester)-nanoparticle mediated transfection of retinal pigment epithelial cells in vitro and in vivo. PLoS ONE. 2012;7(5):e37543. 22629417 10.1371/journal.pone.0037543 PMC3357345

[45] Bhise NS, Wahlin KJ, Zack DJ, Green JJ. Evaluating the potential of poly(beta-amino ester) nanoparticles for reprogramming human fibroblasts to become induced pluripotent stem cells. Int J Nanomed. 2013;8:4641–58. 10.2147/IJN.S53830 PMC3857166 24348039

[46] Vaughan HJ, et al. Polymeric nanoparticles for dual-targeted theranostic gene delivery to hepatocellular carcinoma. Sci Adv. 2022;8:1–13. 10.1126/sciadv.abo6406 PMC9299552 35857843

[47] Cutlar L, et al. Highly branched poly(β-Amino Esters): synthesis and application in Gene Delivery. Biomacromolecules. 2015;16:2609–17. 26265425 10.1021/acs.biomac.5b00966

[48] Durán-Mota JA, Yani JQ, Almquist BD, Borrós S, Oliva N. Polyplex-loaded hydrogels for local gene delivery to human dermal fibroblasts. ACS Biomater Sci Eng. 2021;7(9):4347–61. 10.1021/acsbiomaterials.1c00159. Epub 2021 Jun 3; PMID: 34081451. 34081451 10.1021/acsbiomaterials.1c00159

[49] Bingol B, et al. One-step injectable and bioreducible Poly(β-Amino Ester) hydrogels as controlled drug delivery platforms. ACS Appl Polym Mater. 2019;1:1724–34.

[50] Kim J, et al. Poly(ethylene glycol)–Poly(beta-amino ester)-based nanoparticles for suicide gene therapy enhance brain penetration and extend survival in a preclinical human glioblastoma orthotopic xenograft model. ACS Biomater Sci Eng. 2020;6:2943–55. 33463272 10.1021/acsbiomaterials.0c00116 PMC8035708

[51] Kim J, et al. Verteporfin-loaded poly(ethylene glycol)-Poly(beta-amino ester)-Poly(ethylene glycol) triblock micelles for cancer therapy. Biomacromolecules. 2018;19:3361–70. 29940101 10.1021/acs.biomac.8b00640 PMC6249031

[52] González-Ríos N, et al. Novel α-mannose-functionalized poly(β-amino ester) nanoparticles as mRNA vaccines with increased antigen presenting cell selectivity in the spleen. J Mater Chem B. 2023;11:6412–27. 37350113 10.1039/d3tb00607g

[53] Fornaguera C, et al. mRNA delivery system for targeting antigen-presenting cells in vivo. Adv Healthc Mater. 2018;7:e1800335. 29923337 10.1002/adhm.201800335

[54] Fornaguera C, et al. Engineering oncogene-targeted anisamide-functionalized pBAE nanoparticles as efficient lung cancer antisense therapies. RSC Adv. 2023;13:29986–30001. 37842686 10.1039/d3ra05830a PMC10573942

[55] Cosialls R, et al. Ammonium trifluoroborate-modified poly(β-aminoesters): a case study for PET-guided in vivo pharmacokinetic studies of a non-viral gene delivery system. J Control Release. 2023;358:739–51. 37207793 10.1016/j.jconrel.2023.05.017

[56] Fornaguera C, Castells-Sala C, Lázaro MA, Cascante A, Borrós S. Development of an optimized freeze-drying protocol for OM-PBAE nucleic acid polyplexes. Int J Pharm. 2019;569:118612. 31415876 10.1016/j.ijpharm.2019.118612

[57] Fornaguera C et al. Synthesis and characterization of mRNA-loaded poly (beta aminoesters) nanoparticles for Vaccination purposes. J Vis Exp. 2021. 10.3791/62889. 10.3791/62889 34459811

[58] Riley RS, June CH, Langer R, Mitchell M. J. Delivery technologies for cancer immunotherapy. Nat Rev Drug Discov. 2019;18:175–96. 30622344 10.1038/s41573-018-0006-z PMC6410566

[59] Fornaguera C, et al. In vivo retargeting of poly(beta aminoester) (OM-PBAE) nanoparticles is influenced by Protein Corona. Adv Healthc Mater. 2019;8(19):1900849. 10.1002/adhm.201900849. 10.1002/adhm.201900849 31478348

[60] González-Ríos N, et al. Novel α-mannose-functionalized poly(β-amino ester) nanoparticles as mRNA vaccines with increased antigen presenting cell selectivity in the spleen. J Mater Chem B. 2023. 10.1039/d3tb00607g. 37350113 10.1039/d3tb00607g

[61] Green JJ, et al. Electrostatic ligand Coatings of nanoparticles enable ligand-specific gene delivery to human primary cells. Nano Lett. 2007;7:874–9. 17362046 10.1021/nl062395b

[62] Smith TT, et al. In situ programming of leukaemia-specific T cells using synthetic DNA nanocarriers. Nat Nanotechnol. 2017;12:813–20. 28416815 10.1038/nnano.2017.57 PMC5646367

[63] Dobrovolskaia Ma, McNeil SE. Handbook of immunological properties of engineered nanomaterials. Front Nanobiomed Res. 2013. 10.1038/nnano.2007.223 18654343

[64] García-Fernández C, Saz A, Fornaguera C, Borrós S. Cancer immunotherapies revisited: state of the art of conventional treatments and next-generation nanomedicines. Cancer Gene Ther. 2021. 10.1038/s41417-021-00333-5. 33837365 10.1038/s41417-021-00333-5

[65] Dold NM, Zeng Q, Zeng X, Jewell CM. A poly(beta-amino ester) activates macrophages independent of NF-κB signaling. Acta Biomater. 2018;68:168–77. 29292166 10.1016/j.actbio.2017.12.040 PMC6292427

[66] Brugada-vilà P, et al. Oligopeptide-modified poly (beta-amino ester) s–coated AdNuPARmE1A: boosting the efficacy of intravenously administered therapeutic adenoviruses. Theranostics. 2020;10(6):2744. 32194832 10.7150/thno.40902 PMC7052890

[67] Balashanmugam MV, et al. Preparation and characterization of novel PBAE/PLGA polymer blend microparticles for DNA vaccine delivery. Sci World J. 2014;2014:385135. 10.1155/2014/385135. 10.1155/2014/385135 PMC4225858 25401137

[68] Ben-Akiva E, et al. Biodegradable lipophilic polymeric mRNA nanoparticles for ligand-free targeting of splenic dendritic cells for cancer vaccination. Proc Natl Acad Sci USA. 2023;120(26):e2301606120. 10.1073/pnas.2301606120. Epub 2023 Jun 20. 37339211 10.1073/pnas.2301606120 PMC10293809

[69] Luly KM, Green JJ, Sunshine JC, Tzeng SY. Biomaterial-mediated genetic reprogramming of merkel cell carcinoma and melanoma leads to targeted cancer cell killing in vitro and in vivo. ACS Biomater Sci Eng. 2023;9:6438–50. 37797944 10.1021/acsbiomaterials.3c00885 PMC10646862

[70] Liu Y, Li Y, Keskin D, Shi L. Poly(β-Amino Esters): synthesis, formulations, and their biomedical applications. Adv Healthc Mater. 2019;8:1–24. 10.1002/adhm.201801359 30549448

[71] Wang QT, et al. Tumor-associated antigen-based personalized dendritic cell vaccine in solid tumor patients. Cancer Immunol Immunother. 2020;69:1375–87. 32078016 10.1007/s00262-020-02496-w PMC11027674

[72] Verbeke R, et al. Co-delivery of nucleoside-modified mRNA and TLR agonists for cancer immunotherapy: restoring the immunogenicity of immunosilent mRNA. J Control Release. 2017;266:287–300. 28987878 10.1016/j.jconrel.2017.09.041

[73] Kauffman KJ, et al. Efficacy and immunogenicity of unmodified and pseudouridine-modified mRNA delivered systemically with lipid nanoparticles in vivo. Biomaterials. 2016;109:78–87. 27680591 10.1016/j.biomaterials.2016.09.006 PMC5267554

[74] Persano S, et al. Lipopolyplex potentiates anti-tumor immunity of mRNA-based vaccination. Biomaterials. 2017;125:81–9. 28231510 10.1016/j.biomaterials.2017.02.019 PMC5378555

[75] Borrós S, Fornaguera C, Garcia-Fernandez C. Zwitterionic functionalized Poly(Beta-aminoester) polymers and uses thereof. 2021.

[76] Zou C, et al. Targeted co-delivery of Trp-2 polypeptide and monophosphoryl lipid A by pH-sensitive poly (β-amino ester) nano-vaccines for melanoma. Nanomedicine. 2019;22:102092. 10.1016/j.nano.2019.102092. Epub 2019 Oct 5; PMID: 31593795. 31593795 10.1016/j.nano.2019.102092

[77] Wang S, et al. Viral vectored vaccines: design, development, preventive and therapeutic applications in human diseases. Signal Transduct Target Ther. 2023. 10.1038/s41392-023-01408-5. 37029123 10.1038/s41392-023-01408-5 PMC10081433

[78] Chen L, Zuo M, Zhou Q, Wang Y. Oncolytic virotherapy in cancer treatment: challenges and optimization prospects. Front Immunol. 2023;14:1308890. 38169820 10.3389/fimmu.2023.1308890 PMC10758479

[79] Andorko JI, Pineault KG, Jewell CM. Impact of molecular weight on the intrinsic immunogenic activity of poly(beta amino esters). J Biomed Mater Res A. 2017;105:1219–29. 27977902 10.1002/jbm.a.35970

[80] Justilien V, Fields AP. Utility and applications of orthotopic models of human non-small cell lung cancer (NSCLC) for the evaluation of novel and emerging cancer therapeutics. Curr Protoc Pharmacol. 2013;62(1):14–27. 10.1002/0471141755.ph1427s62 PMC4362678 24510718

[81] Oh S, Wilcox M, Pearson J, Borrós S. Optimal design for studying mucoadhesive polymers interaction with gastric mucin using a quartz crystal microbalance with dissipation (QCM-D): comparison of two different mucin origins. Eur J Pharm Biopharm. 2015;96:477–83. 26272125 10.1016/j.ejpb.2015.08.002

[82] Riera R, et al. Tracking DNA complexation state of pBAE polyplexes in cells with super resolution microscopy. Nanoscale. 2019;11(38):17869–77. 31552987 10.1039/c9nr02858g

[83] Dosta P, Ramos V, Borrós S. Stable and efficient generation of poly(β-amino ester)s for RNAi delivery. Mol Syst Des Eng. 2018;3:677–89.

[84] Dosta P, et al. Delivery of anti-microRNA-712 to inflamed endothelial cells using poly(β-amino ester) nanoparticles conjugated with VCAM-1 targeting peptide. Adv Healthc Mater. 2021;2001894:1–11. 10.1002/adhm.202001894 PMC8277885 33448151

[85] Puigmal N, et al. Microneedle-based local delivery of CCL22 and IL-2 enriches Treg Homing to the skin allograft and enables temporal monitoring of Immunotherapy Efficacy. Adv Funct Mater. 2021;31:1–12.

[86] Guerra-Rebollo M, et al. Electrostatic coating of viral particles for gene delivery applications in muscular dystrophies: influence of size on stability and antibody protection. J Neuromuscul Dis. 2021;8:815–25. 34366365 10.3233/JND-210662

[87] Riera R, Tauler J, Feiner‐Gracia N, Borrós S, Fornaguera C, Albertazzi L. Complex pBAE nanoparticle cell trafficking: tracking both position and composition using super resolution microscopy. ChemMedChem. 2022;17(13):e202100633. 10.1002/cmdc.202100633. Epub 2022 Mar 18. 35212466 10.1002/cmdc.202100633 PMC9400995

[88] Sadeqi Nezhad M. Poly (beta-amino ester) as an in vivo nanocarrier for therapeutic nucleic acids. Biotechnol Bioeng. 2023;120:95–113. 36266918 10.1002/bit.28269

[89] Kaczmarek JC, Kowalski PS, Anderson DG. Advances in the delivery of RNA therapeutics: from concept to clinical reality. Genome Med. 2017;9:1–16. 28655327 10.1186/s13073-017-0450-0 PMC5485616

[90] Warminski M, Mamot A, Depaix A, Kowalska J, Jemielity J. Chemical modifications of mRNA ends for therapeutic applications. Acc Chem Res. 2023;56:2814–26. 37782471 10.1021/acs.accounts.3c00442 PMC10586375

[91] Segovia N, Pont M, Oliva N, Ramos V, Borrós S. A. N. Hydrogel doped with nanoparticles for local sustained release of siRNA in breast cancer. Adv Healthc Mater. 2015;4:271–80. 25113263 10.1002/adhm.201400235

[92] Tzeng SY, Hung BP, Grayson WL, Green JJ. Cystamine-terminated poly(beta-amino ester)s for siRNA delivery to human mesenchymal stem cells and enhancement of osteogenic differentiation. Biomaterials. 2012;33:8142–51. 22871421 10.1016/j.biomaterials.2012.07.036 PMC3432640

[93] Tzeng SY, Green JJ. Subtle changes to polymer structure and degradation mechanism enable highly effective nanoparticles for siRNA and DNA delivery to human brain cancer. Adv Healthc Mater. 2013;2:468–80. 23184674 10.1002/adhm.201200257 PMC3838886

[94] Kozielski KL, Tzeng SY, Green JJ. A bioreducible linear poly(β-amino ester) for siRNA delivery. Chem Commun. 2013;49:5319–21. 10.1039/c3cc40718g PMC3894248 23646347

[95] Zhou D, et al. Highly branched poly(β-amino ester)s for skin gene therapy. J Control Release. 2016;244:336–46. 27288877 10.1016/j.jconrel.2016.06.014

[96] Niu G, et al. An effective vaginal gel to deliver CRISPR/Cas9 system encapsulated in poly (β-amino ester) nanoparticles for vaginal gene therapy. EBioMedicine. 2020;58:1–12. 10.1016/j.ebiom.2020.102897 PMC7387785 32711250

[97] Rui Y, et al. Poly(Beta-Amino Ester) nanoparticles enable nonviral delivery of CRISPR-Cas9 plasmids for gene knockout and gene deletion. Mol Ther Nucleic Acids. 2020;20:661–72. 32380416 10.1016/j.omtn.2020.04.005 PMC7210380

[98] Green JJ, Kozielski K, Tzeng SY. Bioreducible poly (Beta-amino ester) s for sirna delivery. 2013. 10.1007/978-1-4939-3112-5_8 PMC4745093 26472444

[99] Bauche C, Vaillant R, Sarry E, Mourlane F, Bishop P. Polymer-encapsulated viral vectors for genetic therapy. 2019.

[100] Ozgul T, Mourlane F, Vaillant F, Bauche C. Targeted poly(beta aminoesters). 2022. https://patents.google.com/patent/AU2020349085A1/en.

[101] Popel AS, Karagiannis ED. Peptide modulators of angiogenesis and uses thereof. 2013. https://patents.google.com/patent/US8507434B2/en.

[102] Hanes J, Suk JS, Mastorakos P. Highly stable biodegradable gene vector platfomr for overcoming biological barriers. 2010;542. https://patents.google.com/patent/US10335500B2/en.

[103] Salvador Borrós CF, Garcia-Fernandez C. Zwitterionic functionalized poly (beta aminoesters) = polymers and uses thereof. 2021. https://www.patentguru.com/assignee/institut-qu%C3%ADmic-de-sarri%C3%A0-cets-fundaci%C3%B3-privada.

[104] Haifa S. Core-shell structure platform for immunotherapy. 2018;637. https://patents.google.com/patent/WO2018140826A1/en.

[105] Green JJ, Kim J, Tzeng S. Poly (beta aminoester)-co-polyethylene glycol polymers for gene and drug delivery. 2018. https://www.patentguru.com/inventor/green-jordan.

[106] Green JJ et al. Polymeric nanoparticle genetic vaccines. 2021. https://jhu.technologypublisher.com/technology/53106.

[107] Borrós S, Guerra-Rebollo M, Lopez-Pinto MS, Montolio MS. Covalently-coated adeno-assocated virus vector for its use in gene therapy. 2023. https://www.patentguru.com/inventor/borr%C3%B3s-g%C3%B3mez-salvador.

